# The Role of Hedgehog Signaling Pathway in Head and Neck Squamous Cell Carcinoma

**DOI:** 10.3390/cells12162083

**Published:** 2023-08-17

**Authors:** Piotr Cierpikowski, Anna Leszczyszyn, Julia Bar

**Affiliations:** 1 Department of Maxillofacial Surgery, The Ludwik Rydygier Specialist Hospital, Osiedle Zlotej Jesieni 1, 31-826 Krakow, Poland; 2 Dental Surgery Outpatient Clinic, 4th Military Clinical Hospital, Weigla 5, 53-114 Wroclaw, Poland; anna.leszczyszyn@wp.pl; 3 Department of Immunopathology and Molecular Biology, Wroclaw Medical University, Bujwida 44, 50-345 Wroclaw, Poland

**Keywords:** hedgehog, head and neck cancer, signaling pathways, cancer stem cells, molecular targeted therapy

## Abstract

Head and neck squamous cell carcinoma (HNSCC) is the sixth leading malignancy worldwide, with a poor prognosis and limited treatment options. Molecularly targeted therapies for HNSCC are still lacking. However, recent reports provide novel insights about many molecular alterations in HNSCC that may be useful in future therapies. Therefore, it is necessary to identify new biomarkers that may provide a better prediction of the disease and promising targets for personalized therapy. The poor response of HNSCC to therapy is attributed to a small population of tumor cells called cancer stem cells (CSCs). Growing evidence indicates that the Hedgehog (HH) signaling pathway plays a crucial role in the development and maintenance of head and neck tissues. The HH pathway is normally involved in embryogenesis, stem cell renewal, and tissue regeneration. However, abnormal activation of the HH pathway is also associated with carcinogenesis and CSC regulation. Overactivation of the HH pathway was observed in several tumors, including basal cell carcinoma, that are successfully treated with HH inhibitors. However, clinical studies about HH pathways in HNSCC are still rare. In this review, we summarize the current knowledge and recent advances regarding the HH pathway in HNSCC and discuss its possible implications for prognosis and future therapy.

## 1. Introduction

Head and neck squamous cell carcinoma (HNSCC) is the most common head and neck malignancy, with a global incidence of about 890,000 cases annually [1]. HNSCC arises from the upper aerodigestive tract mucosa and is located in sites like the lip, oral cavity, nasopharynx, pharynx, and larynx [1]. The etiology of HNSCC is multifactorial, including tobacco smoking, alcohol abuse, betel chewing, and human papillomavirus (HPV) or Epstein–Barr virus (EBV) infections [2]. Currently, radical surgical resection of tumors in combination with radio/chemotherapy is the standard treatment option that provides limited efficacy in advanced cases. Despite intensive development in oncology in recent years, HNSCC is characterized by a poor prognosis and remains among the most lethal cancers worldwide [1,2]. During the last few decades, the 5-year survival rate of patients with HNSCC has not improved significantly and is estimated at about 60% [3]. In addition, the prevalence of HNSCC is increasing worldwide [1,2]. Progress in treatment is low, and novel targeted therapies for HNSCC are marginal [4]. Up to date, of a wide range of molecular antibodies, only cetuximab, nivolumab, and pembrolizumab are available for the treatment of recurrent or metastatic HNSCC [2,4]. Thus, it is very important to develop novel therapeutic options based on molecular alterations detected in HNSCC. Molecularly targeted therapies are used clinically with success in other tumors like melanoma, lung cancer, and breast cancer [5]. Nonetheless, molecular-targeted therapies are still limited treatment options in HNSCC [6].

Among the many molecular alterations occurring in HNSCC, researchers have recently focused on signaling pathways that normally regulate several processes related to cell proliferation, differentiation, and stemness, while their aberrant activation has been detected in various cancers [6,7]. An especially promising target for novel therapies is the Hedgehog (HH) signaling pathway, which was first detected in 1980 by Christiane Nűsslein-Volhard and Eric Wieschaus during mutation screening in Drosophila melanogaster [8]. Further studies showed that HH signaling plays a crucial role not only in embryogenesis but also in many human disorders, including cancer [9]. Based on these findings, inhibition of the HH pathway was introduced in selected carcinomas, where it is currently used with clinical success [10]. However, reports studying the role of HH signaling in HNSCC are limited, and its clinical significance is still not determined.

In this paper, we aim to summarize the current knowledge and recent advances regarding the HH signaling pathway in HNSCC and discuss its possible relevance in tumorigenesis, progression, and future therapy, indicating new directions for further research.

## 2. HNSCC and Tumorigenesis Model

**Figure 1:**
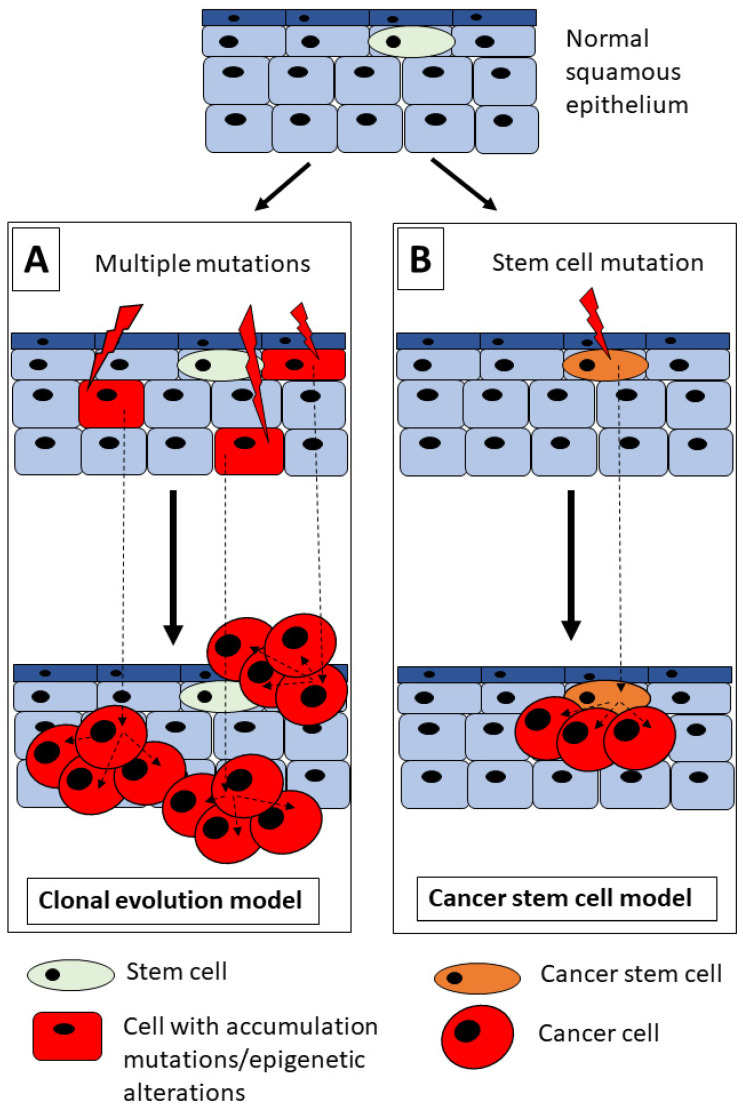
Two models of carcinogenesis for head and neck squamous cell carcinoma: (**A**) In the stochastic model, any tumor cell has equal ability to initiate tumors. (**B**) In the hierarchical or cancer stem cell (CSC) model, only CSCs are able to originate new tumors.

The poor prognosis of HNSCC is caused by the rapid dissemination of tumor cells to cervical lymph nodes, resistance to chemo/radiotherapy, and a high risk of local tumor recurrence [11]. Generally, initial tumors may be cured by a combination of surgery and radio/chemotherapy, but successful therapy is limited proportionally to the clinical stage of the tumor [6]. However, even if the above therapies are combined, a clinical response is observed in nearly 100% of the early tumors but only in one-third of the advanced tumors [6]. The low response of HNSCC to current treatment modalities is associated with their intratumoral heterogeneity, which reflects differences in cell subpopulations existing in the tumor tissue and explains heterogeneous sensitivity to anticancer therapy [11,12]. This heterogeneity of HNSCC seems to be a result of several independent factors like genetic mutations, environmental impact, or intracellular modifications [13]. As shown in Figure 1, two models of HNSCC tumorigenesis have been proposed that may explain the cellular heterogeneity of HNSCC: the clonal evolution model and the cancer stem cell (CSC) model of tumorigenesis [7,13,14]. 

The clonal evolution model of tumor development demonstrated by Peter Nowell suggests that cancer is an evolutionary process induced by the growth and expansion of subclones that carry selectively advantageous genetic alterations [15]. The high genetic instability observed in subclones of cancer cells reflects the accumulation of gene mutations [15]. The clonal evolution model of tumor growth suggests that all cells existing in the tumor have the same potential to form tumors, what may explain the progressive growth and resistance to radio/chemotherapy in selected tumors [15,16]. Cell clonal analyses showed that HNSCC progression might be associated with the clonal evolution model of tumor development [16]. Moreover, a genetic sequence comparison of HNSCC tissues from the primary tumor and metastatic focus showed that there are two different patterns of clonal dynamics [13,17]. Nonetheless, published evidence supports the hierarchical model of solid tumor growth [14]. The proposed CSC model suggests that CSCs are the highest in this hierarchy, symmetrically dividing to complete the CSC population, whereas asymmetric division results in daughter cells with a low potential for tumorigenesis [10,13,16]. CSCs present the same properties as normal tissue stem cells, like self-renewal ability, uncontrolled proliferation, and differentiation [6,13]. A hierarchical organization of tumors is the primary difference between the CSC model and the clonal evolution model observed in many solid tumors, including HNSCC [14,16,18]. Although these models are still under study, it was shown in Figure 1 that these two models are considered for HNSCC development because CSCs might undergo clonal evolution during tumor growth under the influence of external factors like hypoxia, nutrient deficiency, or anticancer therapy [14]. In light of this fact, various CSC populations may exist in a single tumor, and their cellular composition has an impact on cancer progression and resistance to chemo/radiotherapy [10,14,19]. Interestingly, Kreso and Dick revealed that differentiated cancer cells forming a tumor (non-CSCs) might acquire stem cell features and thereby transform into “induced” CSCs [19].

It was hypothesized that some types of cancer cells with unique features have been described as CSCs, which become highly malignant, leading to cancer recurrence, metastasis, and resistance to radio/chemotherapy [6,7,12,20]. It was shown that CSCs are a small subpopulation of cancer cells that share common molecular features and express similar biomarkers to embryonic and normal adult stem cells [6,13]. It was postulated that CSCs within the bulk tumor are resistant to conventional anticancer therapies, leading to tumor recurrence [13]. Interestingly, it was noted that CSCs are not an individual cell population but consist of multiple heterogeneous subclones. It is difficult to define which one contributes to metastasis, tumorigenicity, and resistance to chemo/radiotherapy [6,7,12]. These properties were related to the activation of signaling pathways, such as HH, WNT, NOTCH, PI3K, Hippo, or nuclear factor-kappa B (NF-κB), that control detoxification, activate resistance to apoptosis and autophagy, increase the presence of drug transporter proteins, and regulate stem cell transcription factors [10,18]. Many studies imply that it is essential to expand our knowledge about the signaling pathways related to head and neck CSCs in order to develop novel targeted therapies for HNSCC [6,7,10,13,14,18].

## 3. The HH Signaling Pathway

### 3.1. Overview of HH Signaling

The HH pathway is a strictly conserved evolutionary signaling cascade that controls embryonic development and regulates normal cell growth and differentiation under physiological conditions [21,22]. This pathway also plays a key role in the maintenance of adult stem cells [23]. The HH pathway consists of receptors and ligands that cooperate under this signaling pathway activation [24]. HH is a 45 kDa protein that is the result of the autoproteolytic cleavage of 20 kDa HH ligand precursor proteins. This leads to the production of an N-terminal protein, followed by dual lipid modifications covalently bound to palmitic acid and cholesterol [25]. HH has three gene homologs: Sonic HH (SHH), Desert HH (DHH), and Indian HH (IHH), which express the following proteins: SHH, DHH, and IHH [21,25]. HH proteins are modified post-translationally and released by the secreting cell as HH ligands with the participation of Dispatched (DISP), a transmembrane transporter protein [21,23]. These ligands play distinct roles during embryonic development. SHH is the most widely present HH ligand in vertebrates, and its paracrine effects on adjacent cells are the most common feature of transduction signals. However, HH may also have an autocrine effect [23,24]. SHH is important for neuronal development, whereas IHH is relevant in skeletal development, especially in bone formation. Additionally, DHH is crucial to the gonads’ development [25]. The HH signaling pathway is complex and consists of HH ligands, the 12-transmembrane receptor Patched (PTCH) with two large extracellular loops crucial for HH ligand binding, the 7-transmembrane protein Smoothened (SMO), and the glioma-associated oncogene (GLI) transcriptional factor that is crucial for the production of HH target genes [24,25]. In general, HH signaling might be activated by two different mechanisms: canonical or non-canonical pathways [18,26].

### 3.2. Canonical Pathway

**Figure 2:**
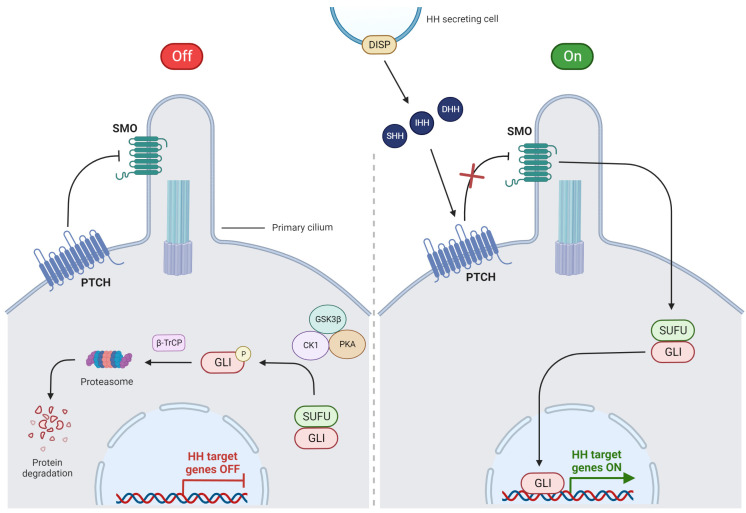
The schematic model of the canonical HH signaling pathway. On the left side, when there is a lack of HH ligand, the PTCH receptor inhibits SMO, which turns off HH signaling, and GLI is degraded in the proteasome. On the right side, when the HH ligand is present, the PTCH receptor stops inhibition of SMO, which allows GLI to translocate into the nucleus and turn on HH signaling.

Activation of HH signaling by the canonical mechanism requires the involvement of the primary cilium with its specialized transmembrane receptors [10]. One of these receptors is PTCH1, which is placed near the primary cilium, specific structure that is exposed on the cell membrane and binds with HH ligands such as SHH, DHH, and IHH [10,27]. When one of these ligands binds to PTCH1, the receptor is internalized, causing accumulation of SMO protein in the primary cilium, which in turn signals the suppressor of fused (SUFU) to release GLI proteins. This permits GLI to translocate into the nucleus in order to start the transcription of HH target genes [10,21,28]. GLI1 is responsible for the genes associated with cell proliferation (cyclins D1, D2, and N-myc) and survival (BCL-2), while GLI2 and GLI3 have repressive functions [10,21,24]. In the absence of HH ligands, the PTCH receptor inhibits SMO protein, and the HH pathway is blocked [10]. SMO is not able to activate GLI, which remains connected with SUFU in the cytoplasm [10]. Accumulating GLI is phosphorylated by a complex of glycogen synthase kinase 3β (GSK3β), casein kinase 1 (CK1), and protein kinase A (PKA) [28]. Then, phosphorylated GLI is detected by β-transducin repeat-containing protein (β-TrCP) and directed to degradation in the proteasome. All processes of the canonical HH pathway are illustrated in Figure 2.

### 3.3. Non-Canonical Pathway

In the non-canonical mechanism of HH signaling, activation of GLI proteins can be performed without the participation of SMO, but this way is not yet fully understood [10]. Up to now, there are three known types of non-canonical HH pathways [21]. In contrast to the canonical pathway, the non-canonical HH pathway is activated without HH ligands but with the inhibition of PTCH1 (type I) and/or SMO (type II) or through the immediate activation of GLI proteins by other signaling pathways, like a “bypass”, independently from the signaling through the PTCH1/SMO complex (type III) [26]. In the non-canonical HH pathway, GLI1 may be activated by various signaling pathways, and this crosstalk between different signaling pathways seems to be responsible for anticancer treatment failure [27,28]. It was observed that breast CSCs with increased HH signaling gain adaptation to hypoxic conditions and promote breast tumor metastasis [27]. Additionally, the HH pathway crosstalks with several signaling pathways like mTOR, MAPK, and AKT, which activate genes involved in cell survival (BCL-2), and this mechanism was described as promoting HNSCC progression [28]. Other target genes involved in the oncogenic role of the HH pathway are genes controlling cell proliferation (cyclins D1, E1, and N-myc), promoting angiogenesis (components of platelet-derived growth factor (PDGF) and vascular epithelial growth factor (VEGF), and cell migration (components of epithelial-mesenchymal transition (EMT)) [6]. The role of HH signaling in tumor invasion and migration has been confirmed in several experimental studies [12,29]. Dysfunction of many signaling pathways, including HH, WNT, NOTCH, JAK/STAT, PI3K/PTEN, and NF-kB, was found in CSCs [26]. Recent reports suggest that dysfunction of various signaling pathways may provide properties typical for CSCs, like self-renewal and differentiation [16,17,21,25]. The precise mechanisms underlying the function of signaling pathways and their relationships in normal stem cells and CSCs have not yet been fully elucidated [30].

## 4. HH Pathway in Head and Neck Tissue Formation

HH signaling is originally involved in the proper development of several organs, including head and neck tissues like teeth, lips, palate, and salivary glands [31]. Therefore, dysfunction of the HH pathway during embryogenesis results in a variety of congenital craniofacial deformities, such as holoprosencephaly, cleft lip and palate, and tooth dysplasia [32]. Of the three HH ligands, the most expressed in humans is SHH, which is pivotal in head and neck tissue formation [32]. 

Orofacial cleft (OFC) is the most common birth defect, with incidence in about 1/500–700 living newborns [33]. Up until now, genetic studies have revealed 24 mutations in PTCH1 in patients with OFC that lead to the loss of PTCH1 functions [33]. Dysregulation of the HH pathway results in incomplete fusion of embryonic facial processes and the creation of cleft lips and palates [34]. An experimental study conducted by Heyne et al. showed that inhibition of HH signaling with vismodegib caused OFC in mice [35].

In teeth, HH signaling plays a key role in physiological morphogenesis by controlling crown and root formation at multiple stages of odontogenesis [36]. It was reported that the inactivation of HH signaling is correlated with tooth malformations. The HH pathway is also considered a regulator of dental stem cells, which may lead to providing new dental therapies based on regenerative medicine [31]. Furthermore, dysregulation of HH signaling is related to the pathogenesis of odontogenic tumors like odontogenic keratocyst (OKC) and ameloblastoma (AB) [37]. Recent reports revealed overexpression of HH pathway components in a high percentage of OKCs and ABs, what indicates the role of HH signaling in the formation of these tumors [38,39]. Based on in vitro studies, these findings suggest the intracystic use of HH inhibitors as an alternative option in the treatment of these tumors [40].

## 5. Aberration of HH Signaling Pathway in HNSCC

The importance of HH signaling in oncogenesis was first described in 1996 in Gorlin–Goltz syndrome, a rare autosomal dominant disease that is related to a high predisposition to OKC, basal cell carcinoma (BCC), medulloblastoma, and rhabdomyosarcoma [41]. A mutation of the PTCH1 gene was identified by two independent research groups in patients suffering from this syndrome [42,43]. Inhibition of the HH pathway in patients with unresectable BCC was introduced to clinical practice in 2012 with good efficacy [44]. Based on these optimistic findings, aberrant activation of components belonging to the HH pathway was observed in the next few years in other tumors like gliomas, breast, lung, gastric, and prostate cancer [45,46,47,48,49]. However, studies about HH signaling in HNSCC are still rare, especially clinical ones.

The first analysis of the HH pathway in HNSCC was conducted by Schneider et al. They compared the expression of HH proteins in skin squamous cell carcinoma (SSCC) and HNSCC [50]. Schneider observed an association between increased expression of SHH and worse overall survival (OS) in patients with HNSCC [50]. Currently, there are several reports regarding the expression of the HH pathway in HNSCC, mostly using the immunohistochemical method. Their findings and clinical correlations with clinicopathological parameters are collected in Table 1. It is known that HH signaling molecules are significantly overexpressed in HNSCC tissue, contrary to normal mucosa [51,52]. Moreover, Gonzalez et al. identified a significant increase in HH protein expression from normal mucosa, through dysplasia, to HNSCC tissue, what indicates that HH signaling is involved in tumor initiation [53]. Several studies have demonstrated that the HH pathway plays a crucial role in tumor growth and invasion in HNSCC. Therefore, increased expression of SHH, PTCH1, SMO, GLI1, GLI2, and GLI3 was detected significantly more frequently in advanced HNSCC tumors [54,55,56,57,58,59,60,61]. In most cases, progressing HNSCC invades the mandible or maxilla, what leads to bone destruction. It was identified that HH signaling may enhance the development of HNSCC by modulating osteoclast differentiation and osteolysis [62,63]. An experimental study performed by Qiao et al. showed that inhibition of the HH pathway by GDC-0449 (an SMO inhibitor) decreased osteolytic activity induced by HNSCC [63]. Likewise, other authors observed an association between HH expression and lymph node invasion, what may confirm the involvement of HH signaling in HNSCC progression and dissemination [54,55,59,60,64]. Interestingly, the methylation of ZIC4, an important regulator of HH signaling, was correlated with lymph node metastasis in OSCC [65]. The significance of the HH pathway in the promotion of metastasis in HNSCC was supported by Fan et al., who observed a correlation between HH pathway components and EMT [60]. Furthermore, overexpression of GLI1, GLI2, PTCH1, and SMO was correlated with a higher histological grade of the tumor, what may suggest that HH signaling plays a key role in HNSCC malignancy [55,58,66]. 

The correlation between the expression of HH proteins and prognosis in patients with HNSCC is still not well established. Most studies revealed that increased SHH, PTCH1, SMO, and GLI1 expression are significantly correlated with worse OS [50,57,59,60,67,68]. Contrary to these results, Enzenhofer et al. observed better OS in HPV-negative HNSCC patients with GLI1 and GLI2 overexpression [69]. Additionally, SHH, GLI1, and SMO overexpression were significantly correlated with tumor recurrence and shorter disease-free survival (DFS) [54,67,68]. However, only two of all the studies were confirmed by multivariate COX regression analysis, indicating that the overexpression of GLI1 and SHH may serve as independent poor prognostic biomarkers in HNSCC [57]. Further studies with larger cohorts are required to define the prognostic significance of the HH pathway components in patients with HNSCC.

**Table 1:** The summary of experimental studies evaluating the clinical significance of Hedgehog pathway components in head and neck squamous cell carcinoma.

HH Protein Overexpression	Clinical Findings	Author, Year, Reference
SHH	Worse OS	Schneider et al., 2011 [50]
	Lymph node metastasis	Fan et al., 2014 [60]
	Advanced clinical stage, lymph node metastasis, tumor recurrence	Huaitong et al., 2017 [54]
	Worse OS, shorter DFS	Noman et al., 2020 [67]
	Lower pT stage	Lu et al., 2020 [58]
	Poor prognosis, worse OS, advanced pT stage	Cierpikowski et al., 2021 [57]
PTCH1	Lymph node metastasis, worse OS	Wang et al., 2012 [59]
	Higher tumor grade	Leovic et al., 2012 [66]
	Advanced pT stage, tumor grade	Lu et al., 2020 [58]
SMO	Lower clinical stage	Cavicchioli Buim et al., 2011 [70]
	Worse OS, shorter DFS	Richtig et al., 2019 [68]
	Advanced pT stage, higher tumor grade	Lu et al., 2020 [58]
	Lymph node metastasis	Schlaepfer Sales et al., 2021 [64]
GLI1	Poor prognosis, regional–distant metastasis	Chung et al., 2011 [71]
	Larger tumor size, tumor recurrence, lymph node metastasis, worse OS	Wang et al., 2012 [59]
	Lower tumor grade	Leovic et al., 2012 [66]
	Advanced clinical stage, lymph node metastasis, worse OS	Fan et al., 2014 [60]
	Better OS	Enzenhofer et al., 2016 [69]
	Advanced clinical stage, lymph node metastasis, tumor recurrence	Huaitong et al., 2017 [54]
	Lymph node metastasis, higher tumor grade	Chen et al., 2018 [55]
	Advanced clinical stage	Dantas et al., 2021 [61]
GLI2	Better OS, longer DFS	Enzenhofer et al., 2016 [69]
	Advanced clinical stage, higher tumor grade	Chen et al., 2018 [55]
GLI3	Advanced pT stage	Rodrigues et al., 2018 [56]

Abbreviations: OS—overall survival, DFS—disease-free survival.

## 6. CSCs in Head and Neck Cancer

**Figure 3:**
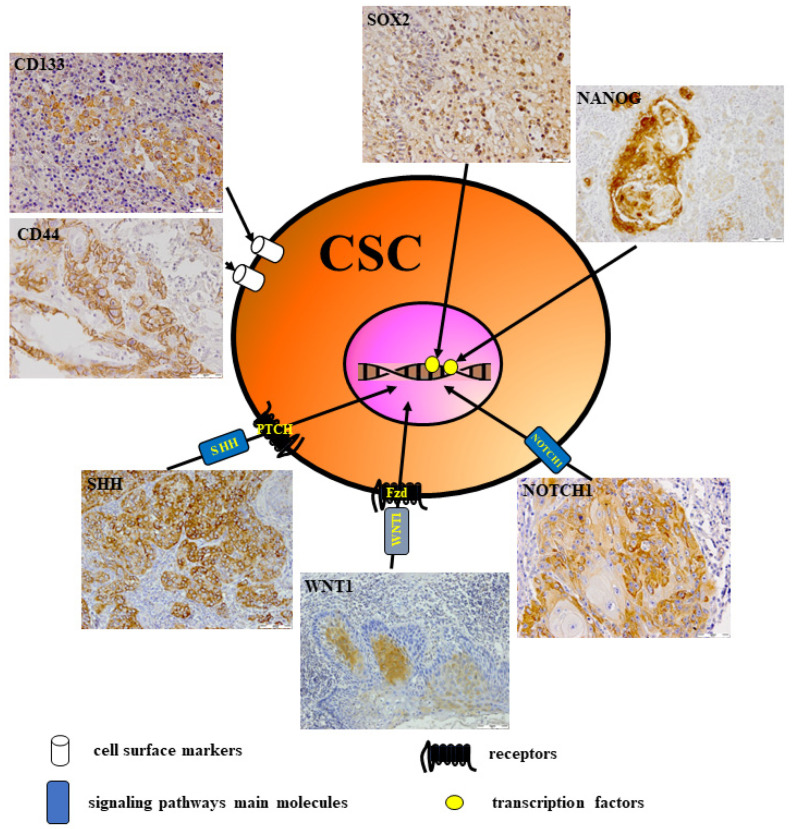
The schematic view of cancer stem cells (CSCs) in head and neck squamous cell carcinoma tissue with expression of main cell surface markers (CD44 and CD133), transcription factors (SOX2 and NANOG), and selected molecules belonging to crucial signaling pathways (SHH, WNT1, and NOTCH1). Immunohistochemical images were adapted from our previous studies [88,89].

The key reason for the aggressive clinical course of head and neck cancer (HNC) is tumor heterogeneity, which reflects differences within cell subpopulations existing in the tumor tissue [6,7]. It was hypothesized that in HNC, there is a specific type of cancer cells with unique features, defined as CSCs. CSCs are proposed as a promising target for HNC therapy. They may also be a valuable biomarker to detect HNC patients with resistance to therapy or a high risk of tumor recurrence [7,72]. CSCs have been identified as a small population of cancer cells in tumor tissue in terms of 0.01–2% of total tumor mass that have similar features to embryonic and normal stem cells [6,73]. CSCs have asymmetrical cell division and self-renewal capability, which induce a heterogeneous subpopulation of cancer cells involved in angiogenesis and immortality [6,7,73]. It is well known that CSCs are more malignant than other subsets of cancer cells in tumor masses. This heightened malignancy contributes to cancer recurrence, metastases, resistance to chemo/radiotherapy, and quick adaptation to variations in the tumor microenvironment, like nutrient deficiency, hypoxia, or increased DNA repair capacity, thereby avoiding cell apoptosis [6,7,74]. It was found that oncogenic mutations accumulated in somatic stem cells induce the transformation of normal stem cells into CSCs [6,73]. The CSC population has been identified in acute myeloid leukemia (AML) as cells showing CD34 and CD38 expression or co-expression of both markers, CD34/CD38. These cells are able to develop AML when inoculated into immunocompromised mice [75]. A few years later, CSC populations were also found in solid tumors [76,77]. Afterward, CSCs have been widely investigated and identified in various types of solid tumors, including HNC, but their role has not been fully described [6,7,76,78]. Several investigators showed that putative CSCs could be isolated from HNSCC tissue by their unique surface biomarker pattern expression [7,73,76,79]. There are several available techniques for isolating CSCs from tumor tissue [7,72,73,76,80]. Generally, CSCs are separated by the detection of combined specific biomarkers that are located on the normal or CSC surface [6,7,24,81,82,83]. Fluorescence-activated cell sorting (FACS) and magnetic-activated cell sorting (MACS) were the first techniques used for CSC separation, which allow multi-biomarker sorting of stem cells at the same time with high purity and strong specificity [80,84]. Other methods used for CSC separation from tumors are based on cell culture with Hoechst 33342 or the colony-forming ability of CSCs [7,24,78]. So far, multiple markers, independently or in combination, have been documented by many authors as putative CSC markers in various tumors, including HNC [6,7,72,73,76,79]. These lists of putative CSC markers include specific biomolecules such as surface markers, drug transporters, enzymes, and components of several signaling pathways [7,72,73,76,78,81]. The majority of these biomarkers are cell surface and cytoplasmic proteins, sometimes located in nuclei. Therefore, they can be detected by antibodies using flow cytometry or immunohistochemistry [78,81,85]. In many solid tumors, including HNSCC, CSCs have been isolated and identified by the detection of surface proteins such as CD44, CD133, CD90, and CD117 [7,73,76,78,79,81,82,83]. It was noted that expression of stem cell surface biomarkers, e.g., CD133, CD44, CD24, and CD117, is associated with metastasis to lymph nodes, poor prognosis, and resistance to chemo/radiotherapy in patients with HNSCC [76,79,83,86]. Recently, Yu and Cirillo summarized the major CSC markers in HNC. They described evidence for several well-accepted markers of stemness, including CD44, aldehyde dehydrogenase 1 (ALDH1), SOX2, OCT3/4, and NANOG, as reliable means of identifying and isolating CSCs [87]. However, up to now, no specific biomarker of CSC has allowed for the isolation of a pure CSC population from HNSCC, what confirms the fact that the population of CSCs is very heterogeneous [7,73,78,79]. As presented in Figure 3, HNSCC tissue shows expression of different markers that are described as biomarkers expressed by stem cells, as confirmed in our previous studies [88,89].

CD44 is an adhesive, transmembrane glycoprotein located on the surface of normal and HNSCC cells as a receptor that is involved in extracellular matrix (ECM) communication [6,7,78]. In cancer, CD44 participates in cell migration, motility, survival regulation, and stemness maintenance [7]. The presence of CD44 has also been observed in normal epithelial tissue. However, overexpression of CD44 in HNSCC tissue was associated with a high rate of CSC proliferation, self-renewal, and metastasis [90,91]. Recent data showed that CD44 might exist in different variants, playing a distinct biological role in cells, but only its variant, CD44v3, is overexpressed in CSCs and is related to HNSCC progression [86,91]. Similarly, CD44v6 expression was correlated with tumor grade, lymph node invasion, and a worse clinical outcome in HNSCC patients [84]. Therefore, CD44 expression has been considered by some authors as a possible prognostic biomarker in patients with HNSCC [91,92,93]. Another cell surface marker expressed on CSCs in HNSCC is the glycoprotein CD24, which decreases cell adhesion and increases carcinoma cell metastasis [94]. It was noted that CD24+ cells promoted angiogenesis and showed high resistance to chemotherapy in HNSCC [7,78,95,96].

The ALDH proteins are cytosolic enzymes that participate in numerous physiological and pathological processes in embryonic and adult tissue, including tumorigenesis [7,97]. The presence of ALDH1 in CSCs was observed in co-expression with other related markers, such as MMP-9 and SNAIL, what confirms the relationship between ALDH1 and EMT [97,98]. ALDH1+ cells found in HNSCC tissue defined a subset of tumors showing a more tumorigenic phenotype with higher resistance to radio/chemotherapy [97]. Increased expression of ALDH1 in HNSCC tissue samples is correlated with poor prognosis in patients [6,7,78]. The activity of the ALDH1 isoenzymes was markedly upregulated in head and neck CSCs, and ALDH1 was considered a specific CSC biomarker, mainly when co-expressed with CD44 [78,93]. It was observed that ALDH+ cells influence the behavior of oral CSCs and promote a high capability of tumor formation, increasing invasion and chemo/radiotherapy resistance [72]. 

CD133 expression was detected in normal stem cells and CSCs [6,7,78]. The presence of CD133 was correlated with the high tumorigenicity and invasiveness of HNSCC [6,7,98]. It was found that CD133 overexpression in HNSCC is correlated with tumor grade, lymph node metastasis, and a worse clinical outcome in patients [99,100]. The presence of CD133 correlated with increased proliferation of cells, an EMT phenotype, CSC-sphere formation, and increased in vivo tumorigenicity [74]. CD133+ cell populations showed an increased ability to migrate, invade, form colonies, and resist therapy when compared with CD133-negative populations [98]. It is postulated that CD133 expression might be used as a valuable prognostic biomarker for survival in HNSCC patients [97,98]. Interestingly, it was reported that tumors positive for both CD133 and CD44 showed a more aggressive phenotype when compared to tumors positive for only CD133 or CD44 expression [78].

Head and neck CSCs express the same profile of proteins as those that regulate embryonic stem cells (ESCs), in particular OCT4, SOX2, and NANOG, which were also reported to be expressed in various cancer tissues and were correlated with poor prognosis [7,72,73,76]. These proteins are considered to be specific regulators that determine the stemness properties of CSCs and have an impact on cell behavior, such as self-renewal ability, inhibition of apoptosis, and cell motility [97].

OCT4 (octamer-binding transcription factor 4) has been involved in tumor development, invasion, and metastasis of many tumors, including OSCC [7,73,78]. OCT4 has been overexpressed in various tumors, such as HNSCC, lung, breast, liver, and ovarian cancer [7,73]. High OCT4 expression is associated with a poor survival rate of OSCC and enhanced OSCC progression [6,7,78]. Overexpression of OCT4 was found more frequently in recurrent or metastatic OSCC, what suggests that OCT4 may be a potential marker of aggressive OSCC [95,97]. Growing evidence indicates that OCT4 may be an independent prognostic marker for HNSCC based on its correlation with disease progression [7,78,95,98].

NANOG (Nanog Homeobox) has the ability to inhibit differentiation by maintaining ESC pluripotency, but its overexpression was also observed in HNSCC [73,76,79]. The higher expression of NANOG was reported in precancer and cancer tissue, contrary to the normal mucosa, what suggests its role in HNSCC tumorigenesis [7,73]. Recent data showed that NANOG could not only promote tumor initiation but also increase the formation of CSC colonies in HNSCC [79]. Furthermore, other reports suggested that overexpression of NANOG enhanced EMT and increased resistance to chemo/radiotherapy in HNSCC [96,98]. NANOG was also noted as a potential prognostic factor for OS in HNSCC [98]. Additionally, the increased expression of NANOG is associated with higher tumor grades in different cancers, including HNSCC [7,73]. There are several reports suggesting that NANOG may serve as a regulating factor in the preservation of CSC stemness [7,78]. 

SOX2 (Sex determining region Y-box 2) is a nuclear transcription factor that plays a crucial role in the development of pluripotent cells [100]. SOX2 induces various signal transduction pathways like tumorigenesis, inhibition of apoptosis, migration, invasion, and resistance to radio/chemotherapy [6,7,88]. Increased transcriptional activity of SOX2 seems to be critical for HNSCC growth and development [78,98]. Furthermore, the silence of SOX2 in head and neck CSCs inhibits their ability to uncontrolled self-renew and tumorigenesis [7]. Chung et al. found that high SOX2 mRNA gene expression in HNSCC indicates that SOX2 might be a prognostic factor for HNSCC patients that can predict the response to radiotherapy [101]. Interesting data were presented by the authors, who showed that co-expression of OCT4 and SOX2 is associated with the initial tumor stage and a longer DFS. It suggests the possibility of a novel targeted therapy option against SOX2 and OCT4 [98,102]. As reported by Fu et al., the downregulation of SOX2 and OCT4 expression in advanced HNSCC indicates that SOX2 and OCT4 overexpression noted in initial tumors might decrease gradually during HNSCC progression [102]. A meta-analysis performed by Fan et al. revealed that the expression of CD133, NANOG, and OCT4 showed prognostic significance in patients with HNSCC [103].

CSCs also possess certain remarkable features that allow them to define their metastatic capabilities, drug resistance, invasiveness, and hierarchical differentiation [7,79]. Several reports noted that adenosine triphosphate (ATP)-binding cassette (ABC) transporters are the main factors in the resistance of CSCs to radio/chemotherapy [6,74,78]. ABC transporters are present in normal cells, but their overexpression in cancer cells increases the chemo/radioresistance of tumors [104]. CSCs efficiently express ABC transporter proteins, such as MDR1/ABCB1, MRP1/ABCC1, and ABCG2, that belong to multidrug resistance proteins and ensure cancer cells’ resistance to anticancer therapy [73,105]. Moreover, it was reported that CSC populations have efflux ability due to the high activity of ABC transporters and ALDH, which decreases oxidative stress and increases resistance to cytotoxic agents [6,24]. Agents inducing DNA damage and apoptosis through chemotherapy or radiotherapy could be inhibited because CSCs protect tumor cells from apoptosis by activating DNA repair [73].

Several signaling pathways in normal stem cells contribute to their proliferation, differentiation, self-renewal, and survival. However, they are abnormally activated or repressed during tumorigenesis and facilitate CSC growth and self-renewal [18,24,90]. These signaling pathways may also induce up- or downstream expression of several genes, including growth factors and apoptotic and anti-apoptotic proteins, in CSCs [24,90]. These signaling pathways are not regulated by a single regulator but by an interwoven network of signaling mediators to regulate CSC growth [18]. The acquisition of CSC features was linked to the activation of various signaling pathways. These pathways include WNT, NOTCH, HH, PI3K, JAK/STAT, Hippo, and NF-κB, which induce resistance to apoptosis, promote the overexpression of drug transporter proteins, and enhance the activation of specific stem cell transcription factors [18]. High activation of several signaling pathways, including WNT, NOTCH, and HH, was observed in our and other studies, what might suggest that CSCs are responsible for resistance to standard therapies in patients with HNSCC [18,88,89,101].

## 7. The Role of HH Signaling in HNSCC Tumorigenesis

**Figure 4:**
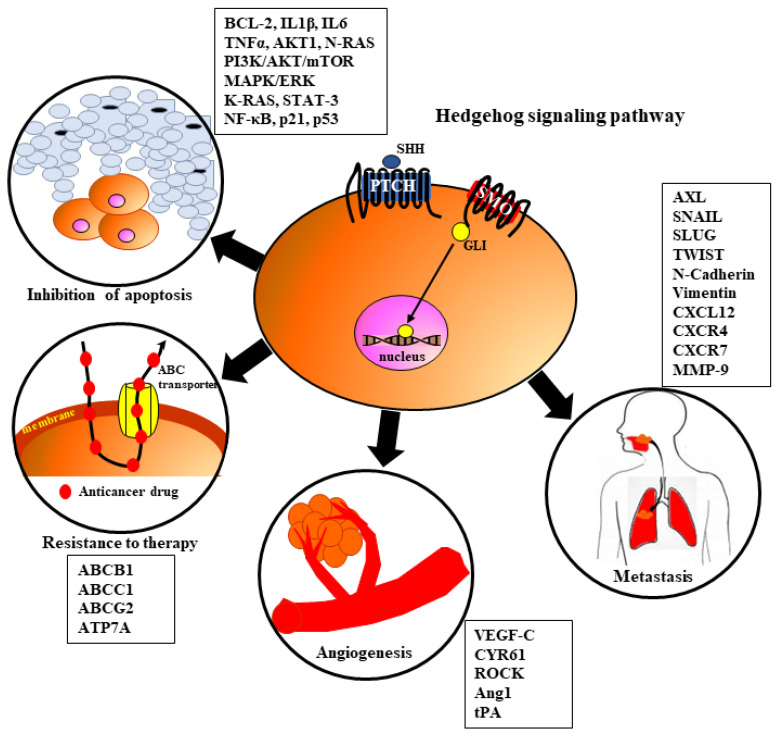
The schematic presentation shows the involvement of Hedgehog signaling in the regulation of crucial processes related to HNSCC tumorigenesis: inhibition of apoptosis, resistance to therapy, angiogenesis, and metastasis by upregulation of different proteins.

Growing evidence revealed that dysregulation of HH signaling is frequently observed in CSCs [7,9,10,13,18]. Several authors postulated that HH, directly and indirectly, has an impact on the biological behavior of many cancers, including HNSCC, by influencing various processes related to tumorigeneses like angiogenesis, metastasis, resistance to therapy, and inhibition of apoptosis (Figure 4) [21,22,69,106,107,108,109,110,111]. Overactivation of HH signaling was documented in various solid tumors. According to the published report, dysregulation of the HH pathway is associated with the development of one-third of all cancers [22,112]. Upregulation of any component belonging to the HH pathway induces its overactivation and thereby leads to carcinogenesis [21,22,29,112]. As described earlier, there are proposed three mechanisms of aberrant HH signaling activation that could be considered in HNSCC development: type I—linked with ligand-independent mechanisms of HH signaling; type II—connected with ligand-dependent oncogenic HH signaling in autocrine/juxtracrine mechanisms; and type III—associated with ligand-dependent HH signaling in paracrine/reverse mechanisms [21,22]. During HNSCC development, HH signaling might be involved in tumor progression, promote tumor growth, and protect tumor cells against treatment modalities [21]. Genetic mutations of components belonging to HH signaling are commonly observed in HNSCC [21].

### 7.1. Angiogenesis

Angiogenesis is a key process determining tumor growth and invasion that is balanced by numerous pro-angiogenic and anti-angiogenic factors [113]. Newly formed blood vessels within the tumor support its rapid progression with required nutrients, facilitate the dissemination of cancer cells from the primary site, and reduce the concentration of cytotoxic agents in tumor tissue [113,114]. Endothelial cells (ECs) express the VEGF receptor (VEGFR), which is bound by VEGF-A, leading to the activation of many signaling cascades that play an important role in cell migration and ECM remodeling [115,116]. It is well known that HH, NOTCH, PI3K/AKT, and MAPK/ERK pathways are involved in the activation of ECs to promote angiogenesis in HNSCC [115,116,117]. As presented in Figure 4, HH signaling induces ECs and surrounding tissue to release many proangiogenic proteins (Ang1, Ang2, and VEGF) [116,118]. In 1998, Pepicelli et al. [119] described for the first time the involvement of the HH pathway in embryonic vascular formation. Similarly, Geng et al. [120] found that HH signaling affects vasculogenesis and angiogenesis, especially during embryogenesis, but its role in tumor angiogenesis is still undefined. In addition, HH ligands are involved in blood vessel maturation, integrity, and arterial differentiation [121]. Some authors pointed out that molecular and cellular mechanisms responsible for HH activation in angiogenesis are not well understood, and published results are conflicting [118,121]. There are data showing that hyperactivation of the HH signaling pathway promotes tumor angiogenesis. A potential association between activated HH signaling and high mRNA levels of VEGF-C was found in many cancers [118,121,122]. It was reported that HH signaling influences angiogenesis induced by ECs through the Rho/RhoA and Rho kinase (ROCK) pathway [123]. This observation was confirmed by experimental data, which revealed that inhibition of the HH signaling pathway by GDC-0449 and cyclopamine decreases the vascular density of colon cancer and OSCC xenografts in animal models [106,124]. While it was reported that SHH expression in low-HH-DLD-1 xenografts promotes neoangiogenesis by inducing tumor vascularity, tumors implanted in Hhip+ mice present higher angiogenesis [106,118,125]. There are reports showing that EMT and CSCs share the same molecular cascades that are crucial for tumor progression through the migration of tumor cells and the formation of metastases [113]. Additionally, analysis of genes related to EMT and CSCs revealed similarities in gene expression, including the HH pathway [113].

Upactivation of the HH signaling pathway in cancer cells induces GLI1 and SHH overexpression, which increases the expression of proangiogenic factors, including VEGF-A, MMP-2, MMP-9, heparanase, and cysteine-rich angiogenic inducer 61 (Cyr61), in different tumor cells [118,124,126]. It was suggested that the function of ECs might be regulated directly by HH ligands secreted by tumor cells [124,127]. SHH overexpression was observed in OSCC, whereas expression of PTCH1 and GLI1/2 was noted in the vascular cells in the front of the tumor [124,127]. SHH may induce neoangiogenesis by increasing the expression of proangiogenic proteins in tumors (e.g., VEGF-A) or by promoting EC proliferation by GLI1 overexpression [118]. HH ligands are expressed not only by tumor cells but also by ECs, which are present mostly in OSCCs and gliomas [118,124]. Blockade of endothelial Scube2 and SHH suppresses angiogenesis in OSCC [54,128]. Most data concludes in reporting that hyperactivation of signaling pathways in tumors might enhance angiogenesis. However, there are opposite results that found that inhibition of SMO using IPI-926 (saridegib) increased vessel density in pancreatic cancer [107]. Also, another SMO inhibitor, NVP-LDE225 (erismodegib), restored vessel density in pancreatic ductal adenocarcinoma, decreased pericyte coverage, enhanced vessel permeability, and increased the proportion of immature microvessels [129]. Interesting data were demonstrated by Takabatake et al. [117], who showed the new significance of HH signaling in the development of OSCC tumor vasculature. The authors suggested that HH signaling facilitates tumor invasion not only in an autocrine manner when stimulating the proliferation of tumor cells but also in a paracrine manner when inducing neoangiogenesis [117]. They also indicated that PTCH expression was not present in blood vessels within OSCC [117]. Nonetheless, OSCCs with overexpression of SHH presented PTCH-positive tumor blood vessels, which confirms that SHH signaling has autocrine and paracrine effects on neoangiogenesis [117]. Similar mechanisms were identified in pancreatic cancer by other authors. They showed that HH signaling promotes neoangiogenesis indirectly by increasing PTCH1 and GLI1 expression and inhibiting antiangiogenic molecules such as THBS2 and TIMP2. They also found that HH signaling directly promotes neoangiogenesis by inducing GTPases belonging to the RHO family in the expression of VEGF [110].

### 7.2. Metastasis

The mechanisms of metastasis induced by HH signaling are understood. However, there are several reports documenting that the HH pathway induces invasion and metastasis through the activation and interaction between different signaling pathways, including HH [117,118,123]. HH promotes metastasis in various solid tumors. Moreover, high activity of the HH, PI3K/AKT/mTOR, and ERK pathways was reported in advanced HNSCC [117,130]. It was found to be a positive association between GLI1 and MMP-9 expression and an inverse correlation between the expression of SHH/GLI1 and E-cadherin in OSCC, which indicates that SHH/GLI-1 is involved in the progression of OSCC and the formation of metastasis [60]. Other studies revealed the role of GLI3 in OSCC metastasis through increased stemness and cell proliferation [56]. The presence of PTCH1, GLI1, and GLI2 protein expression in the invasive front of OSCC might allow cell migration and facilitate metastasis to distant organs [56]. Moreover, PTCH1 expression was frequently observed in advanced stages of HNSCC and was significantly associated with recurrence in patients with OSCC [59].

There are data showing that high upregulation of the HH pathway mediates the function of GLI1, facilitates the CXCL12-induced migration of cancer cells, and increases the metastatic potential of tumor cells [123]. The crucial role of GLI in EMT activation is related to inducing SNAIL expression, what leads to decreased adhesion between cells and influences migration effectiveness [123]. Analysis of the OSCC microenvironment indicated that SHH has an autocrine effect, triggering OSCC invasion. SHH might modulate parenchyma–stromal OSCC by paracrine effect, and both functions of SHH promote metastasis [117].

Downregulation of the GLI family mediates the activation of EMT, leading to the acquisition of a mesenchymal phenotype by epithelial cells [122,123,131]. The loss of cell-cell adhesion and polarity and the gaining of invasive and migratory properties contribute to the aggressive behavior of cancer cells [122,123,131]. HH signaling promotes EMT by decreasing the expression of E-cadherin and increasing the expression of β-catenin and vimentin, thereby enhancing the migration and metastasis of carcinoma cells, which are regulated by factors like SNAIL, SLUG, and TWIST via GLI [131]. There was a positive correlation between HH signaling and SNAIL and MMP-9 expression, as well as a negative correlation with E-cadherin. This suggests that the HH pathway may play a significant role in the progression of HNSCC by regulating EMT [98]. Such a mechanism has been reported in animal models induced by xenografts of gastric adenocarcinoma. In this context, metastatic cells with high expression of GLI2 undergo rapid proliferation through basement tissues, colonize gastric mucosa, and form tumor islands [98,131].

### 7.3. Resistance to Therapy

The current treatment of patients with HNSCC includes a combination of surgery with radio/chemotherapy, which depends on the tumor location and its clinical stage [83]. About one-third of stage I/II HNSCC patients present good survival rates after surgical resection and chemo or radiotherapy alone [83]. Nonetheless, about two-thirds of HNSCC patients in stages III/IV require a more precise therapeutic strategy [83]. First-line standard chemotherapy contains cisplatin or carboplatin plus 5-fluorouracil (5-FU) and cetuximab, whereas a combined therapy strategy is based on 5-FU, docetaxel, and cisplatin [132,133]. However, the acquisition of chemotherapy resistance is frequently observed in HNSCC patients and leads to tumor recurrence and metastasis [132]. It was reported that there are several mechanisms that allow HNSCC cells to avoid cell death/apoptosis after chemo/radiotherapy. These include DNA repair, drug efflux, apoptosis inhibition, activation of the epidermal growth factor receptor (EGFR), NF-κB, and several signaling pathways [109,111,133]. There are studies indicating that the activity of the HH signaling pathway causes chemo/radioresistance in many tumors, including HNSCC [109,122,134]. Moreover, HH participates in several mechanisms like increased DNA damage repair, EMT, drug efflux, alteration or structural modification of drug target proteins, hypoxia, and reduced apoptosis that induce resistance to therapy [109,135,136]. Several reports found that the upstream and downstream expression of HH components, e.g., GLIs, SHH/IHH, SMO, PTCH1, and SUFU, are involved in the chemo/radioresistance of cancer cells [109,135]. Some evidence implicates that HH is a member of the regulatory mechanism that mediates not only oncogenesis but is also responsible for cancer stem cell resistance to therapy in HNSCC [109,133,135]. Moreover, the association between HH signaling and stemness-related genes, e.g., OCT4 and SOX2, suggests that HH might regulate CSCs’ behavior [137]. This hypothesis was confirmed by other authors, who found a correlation between SHH and SOX2 in OSCC. The subset of tumors with co-expression of both biomarkers showed resistance to standard therapy and a shorter survival time [88].

It is well known that HH signaling controls complex mechanisms of DNA repair machinery in normal and tumor cells [109]. This function of the HH pathway is very important when cisplatin is used in HNSCC patients’ therapy because cisplatin induces DNA damage [109,133,135]. Increased expression of EERC1, a protein associated with nucleotide excision repair (NER), was significantly correlated with resistance to cisplatin in HNSCC patients [138]. Additionally, a new association between HH signaling and DNA repair mechanisms with the participation of O-6-methylguanine-DNA methyltransferase (MGMT) was identified in gliomas [109,139]. Cisplatin therapy may be modulated by epigenetic alterations like hypermethylation of different gene promoters [132]. A similar mechanism of DNA methylation was caused by HH activation [109]. Recent data showed that the promoter CpG islands of the neurofilament light polypeptide (NEFL) gene are involved in resistance to cisplatin therapy in the HNSCC cell line [132]. Initial data presented by Wang et al. [140] showed that HH components like SHH and PTCH1 are expressed in the esophageal cancer tissue of patients with micrometastases after receiving chemoradiotherapy. Based on this observation, the researchers postulated that the HH pathway activation mediates chemo/radiotherapy resistance [140]. Interestingly, expression of SHH, PTCH1, and SMO was significantly increased in HNSCCs that were cured with chemotherapy, in contrast to chemotherapy-naive HNSCCs [58]. Other group results showed that high expression of PTCH1, GLI1, and GLI2 activity was connected with more invasive ability, EMT activity, and increased expression of ABC transporters, what leads to enhanced chemoresistance in many solid tumors [141]. Keysar et al. showed that HNSCC cells that acquired resistance to EGFR inhibition revealed upregulation of GLI1 [142]. On the other hand, authors indicated that HNSCC resistance to cisplatin and cetuximab treatment might result from upregulation of the mesenchymal markers (ZEB2, TWIST1, and SNAIL) by transcription of target genes of the HH pathway, which facilitate acquired resistance [142]. The clear correlation between GLI and ABC transporter proteins such as ABCA2, ABCB1, ABCB4, ABCB7, ABCC2, and ABCG1 was revealed by the use of GLI1 inhibitors, which led to reduced expression of ABC transporters in colorectal cancer cells [143]. The role of HH in the resistance of HNSCC to radiotherapy was confirmed by GLI1 upregulation after irradiation [144]. The association between HH signaling, hypoxia-inducible factor 1-alpha (HIF-1α), and temozolomide (TMZ) treatment was reported in an experimental study [109]. It was documented that hypoxia contributes to resistance to TMZ therapy through HIF-1α/SHH/GLI1-dependent alteration of MGMT [139]. The authors found that GLI mediates the expression of MGMT, which promotes resistance to TMZ [139]. There are results presenting that the HIF-1α/SHH/GLI1 axis is involved in the resistance of HNSCC to therapy and that HIF-1α reduces the response of HNSCC to radiotherapy [78,111,122,145]. There are several pieces of evidence documenting the association between CSCs and their signaling pathways and resistance to chemotherapy [109,113,145]. Head and neck CSCs are able to avoid response to therapy due to their overexpression of ABC drug transporters and activation of several pathways [146]. The NOTCH, HH, WNT/β-catenin, PI3K/AKT/mTOR, TGF-β, and STAT pathways are activated and participate in the drug resistance specific for CSCs [147,148,149]. The CSCs with dysregulated HH signaling induced resistance to chemotherapy when platinum, 5-FU, paclitaxel, and doxorubicin were used [150,151]. Song et al. found that SMO and GLI1 expression in the cisplatin (DDP)-resistant A2780 cells (A2780/DDP) was higher than that in native A2780 cells [134]. It was also reported that HH signaling controls esophageal CSC traits, which may increase the resistance of ESCC to chemotherapy [109]. Some authors found an interaction between the GLI1 transcription factor and the promoter of ABCG2 in gastric CSCs [151]. Similar results were noted by other authors, who found that expression of SHH, GLI2, and ABCB1 was increased in the cisplatin-resistant OC SK-OV-3 cell line. Additionally, decreased ABCB1 expression was noted when GLI2 was inhibited with GANT-61 [152]. Interestingly, Cui et al. revealed that the sensitivity of ESCC to cisplatin was dependent on HH activity, what confirms that HH signaling is a key factor in resistance to anticancer therapy [153]. The authors also found that cisplatin therapy enhanced SHH signaling activation by increasing GLI1 and PTCH1 expression, which normally induces upregulation of ABCB1 to reduce cisplatin enrichment in the ALDH-positive ESCC [153]. The subpopulation of CSCs characterized by high activity of ALDH1 was attributed to the radio- and chemoresistance of CSCs in HNSCC [132].

### 7.4. Inhibition of Apoptosis

Apoptosis is one of the main human defense mechanisms by which tumor cells are eliminated during anticancer treatment [154]. However, inhibition of apoptosis is one of the specific properties used by HNSCC to resist cisplatin [133]. Inhibitor of apoptosis protein (IAP) is able to block apoptosis by inhibiting caspases, thereby decreasing cleaved caspase proteins, what explains the resistance of oral CSCs to apoptosis after irradiation [99,155]. It was observed that resistance mechanisms, including activation of anti-apoptotic proteins like BCL-2 family members, are frequently observed in HNSCC cells, especially in CSCs [155]. Inhibitors of BCL-2 combined with cetuximab and radiotherapy revealed good results in the elimination of CSCs in HNSCC cell lines [155]. The apoptosis resistance was induced by the reduced cleaved caspase protein of oral CSCs after irradiation [155]. In laryngeal carcinoma, co-expression of BMI1 with CD133 showed promotion of proliferation and anti-apoptotic effects [99,156].

## 8. The HH Signaling and Viral Infections

There is rising evidence that various viruses target HH signaling to facilitate its transcription, leading to effective and uncontrolled viral replication [117]. The activation of the HH pathway might be destroyed by different viral infections such as HPV, EBV, influenza A (IAV), hepatitis B virus (HBV), hepatitis C virus (HCV), human immunodeficiency virus (HIV), or Kaposi’s sarcoma herpesvirus (KSHV) [123]. It has been found that new vessels are induced by the PDGF-SHH axis. However, this mechanism might be disturbed by KSHV infection, which utilizes the host PDGF receptor (PDGFR) and generates tumorigenesis [157]. Moreover, it is well known that HBV and HCV are involved in the activation of HH signaling in hepatocellular carcinoma (HCC), what contributes to several processes related strictly to HCC tumorigenesis like EMT, maintenance of CSCs, metastasis, and resistance to therapy [158]. Some authors revealed the aberration of HH signaling by HCV in liver fibrosis and HCC but also proposed the possibility of HH inhibitors as a potential target in future therapy [159,160]. Researchers are still investigating the relationship between HCV and HH signaling components to establish the role of both factors in the pathogenesis of HCC [158]. The association between the HH signaling pathway and HPV/E6 protein was confirmed in the murine model of cervical cancer [161]. It has also been found that HPV oncogenes (E6/E7) might increase GLI overexpression in cervical cancer [161].

It is well documented that some of these viruses have oncogenic potential. They play a crucial role in the etiology of HNSCC. Infection with HPV is increasingly common in oropharyngeal squamous cell carcinoma (OPSCC), especially among young non-smokers from developed countries [2,162]. Likewise, EBV infection is associated with nasopharyngeal carcinoma [1]. However, little is known about the associations between HH signaling and HPV and EBV infections in HNSCC. It was postulated that HPV infection might promote carcinogenesis by modifying the stem cell phenotype and transforming oral epithelial stem cells into oral CSCs [73]. GLI1 was proposed as a potential driver in HPV-negative HNSCC [163]. Overexpression of GLI1 and GLI2 has been considered a prognostic parameter for DFS and OS in HPV-negative HNSCC patients undergoing surgical resection and postoperative radiotherapy [69]. Our previous study revealed that some sets of OSCC with SHH expression showed HPV infection, but the relationship has not been statistically significant [57]. Likewise, Richtig et al. did not reveal any correlations between GLI1, SMO, and p16 expression in the HNSCC cohort [68]. Interestingly, such a correlation between p16 and PTCH1 expression was identified by Leovic et al. in OPSCC [66]. Aberrant activation of HH signaling seems to play a significant role in EBV-induced nasopharyngeal carcinoma [158]. It was reported that HH signaling in epithelial cells is activated by EBV, which results in the overexpression of stemness genes [158]. 

## 9. Crosstalk between HH Signaling and Other Pathways

Nowadays, it is not surprising that cell signaling pathways strictly influence themselves through activation or suppression [164]. The crosslink between HH and other stem cell pathways like WNT and NOTCH physiologically plays a significant role in embryogenesis and tissue homeostasis. However, when dysregulated, it may lead to tumorigenesis [165]. The development of HNSCC is a multifactorial and multistep process that is related to the aberration of not only HH signaling but also several other pathways [25,166,167]. In this context, inhibition of only one selected signaling pathway in anticancer treatment may be insufficient, what explains rapid tumor recurrence after an initial good clinical response to the therapy of HNSCC [164].

Despite the fact that the crosstalk between HH and other pathways is known, it is still not fully elucidated. HH signaling is closely related to the WNT pathway by producing WNT ligands like WNT2b, WNT4, and WNT7a, which are required for activation of the WNT pathway [164]. It was noted that GLI1-3 proteins and SUFU may regulate WNT signaling by influencing the accumulation of β-catenin [168,169,170]. Recently, GSK-3β was shown to be important for both HH and WNT signaling [171]. In turn, the WNT pathway produces GLI, which is an essential protein for HH signaling [164]. β-catenin was found to influence GLI stability, thereby enhancing or suppressing the HH pathway [172,173].

The previous genetic analysis identified that NOTCH signaling modulates HH pathway activity by inducing key proteins belonging to the HH pathway, like GLI2, GLI3, and SMO [174]. Several studies reported that molecules of NOTCH signaling, such as Jag1, NICD, Maml1, RBPjK, and NOTCH targets like Hes1, may regulate the HH pathway by interaction with the GLI protein [164,175,176,177]. In addition, NOTCH signaling was identified as a key modulator of the HH pathway by modulation of intracellular transport in the primary cilium, which is a crucial structure for the HH cascade [178]. In turn, the HH pathway controls NOTCH signaling through the expression of its ligands [164]. The close relationship between HH and NOTCH pathways may confirm the fact that HH mutations (mostly PTCH1) were identified recently in T-cell lymphoblastic leukemia, which is associated with NOTCH alteration [179].

A better understanding of this complex crosstalk between signaling pathways involved in tumorigenesis is crucial for developing novel targeted therapies for HNSCC. Simultaneous targeting of several signaling pathways may provide more effective treatment for patients with HNSCC, but additional studies are necessary to confirm this hypothesis.

## 10. Targeting the HH Pathway in HNSCC

### 10.1. Characterization of HH Inhibition

The proven significance of HH signaling in a variety of human tumors has led researchers to target this pathway in anticancer therapy. The first discovered HH inhibitor was cyclopamine, which is a steroidal alkaloid that naturally originated from the corn lily [10]. The possible association between corn lily and blockade of HH signaling was observed in sheep that gave birth to lambs with cyclopia [28]. The discovery of cyclopamine contributed to further studies regarding HH signaling. Nonetheless, cyclopamine is not currently used in treatment due to its low solubility and high toxicity [10]. During the last two decades, over 50 HH inhibitors that block signaling pathways at different steps have been introduced into preclinical or clinical studies [9]. Some of them are currently used in the treatment of various tumors. Interestingly, several natural compounds like curcumin and resveratrol inhibit HH signaling [180,181].

**Figure 5:**
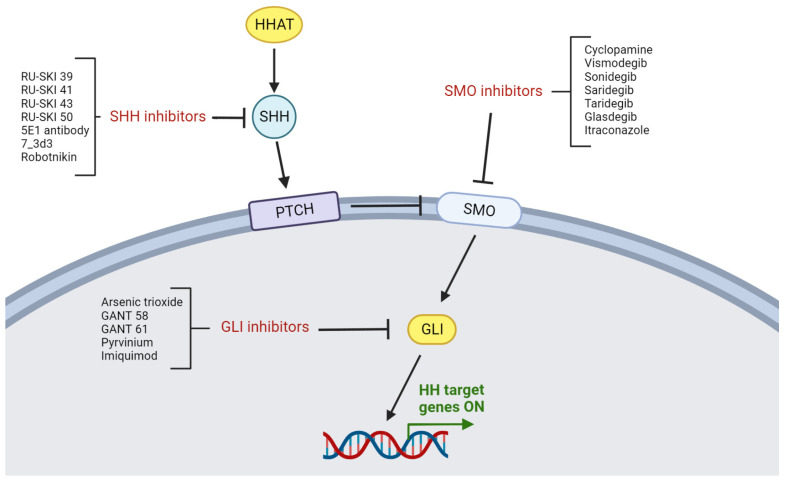
The potential therapeutic approaches for inhibition of the Hedgehog signaling pathway.

As described in Figure 5, the blockade of HH signaling may be performed with the use of inhibitors against specific proteins at each stage of the HH cascade. Based on the above, inhibitors of the HH pathway may be classified as SHH, SMO, and GLI inhibitors.

### 10.2. SHH Inhibitors

HH ligand inhibitors block the binding of HH ligands to the PTCH receptor, thereby inhibiting HH signaling at the first stage of this complex cascade. It is well-known from various tumors that SHH overexpression is associated with increased HH signaling [27]. Therefore, inhibition of HH ligands may be an attractive target that is currently evaluated in preclinical studies in other tumors [44]. However, there have been no published reports yet on the use of SHH inhibitors in HNSCC.

### 10.3. SMO Inhibitors

The most common target for HH inhibitors is the SMO protein. The breakthrough moment in studies regarding HH signaling was the acceptance of vismodegib in 2012 by the Food and Drug Administration (FDA) for treating unresectable BCC [44]. In the next few years, the FDA also approved the use of sonidegib in patients with BCC and glasdegib in patients with AML [10]. Based on this success, many SMO inhibitors were developed and studied in various tumors, including HNSCC. Nonetheless, one of the crucial difficulties in anticancer therapy with SMO inhibitors is the development of drug resistance, which limits their long-term efficacy [9,10].

The positive effect of HH inhibition in HNSCC was first observed by Mozet et al., who studied the influence of cyclopamine, cisplatin, and docetaxel on HNSCC cell colony formation [182]. It was reported that HH inhibition by cyclopamine suppresses colony formation in HNSCC. Furthermore, cyclopamine may increase the chemosensitivity of HNSCC cells when combined in therapy with cisplatin or docetaxel [182]. Similar observations were noted by Gan et al., who revealed that inhibition of HH signaling with cyclopamine enhances HNSCC sensitivity to radiotherapy [183]. The influence of HH inhibition on radiotherapy sensitization in HNSCC was confirmed by Hehlgans et al. [184]. Vismodegib downregulated the HH pathway and sensitized HNSCC cells to radiotherapy [184]. Additionally, it was found that vismodegib decreases the expression of GLI1 in HNSCC cell lines [184]. Recently, the use of vismodegib and itraconazole was also studied in OSCC cell lines, which induced apoptosis and altered the morphology of tumor cells [185]. Based on the encouraging results of the in vitro study, Freitas et al. suggested that SMO inhibitors, such as vismodegib and itraconazole, may be considered for potential future OSCC therapy. Therefore, further clinical trials of HH inhibitors in OSCC patients are required [185].

Downregulation of the HH pathway in HNSCCs after the use of SMO inhibitors was represented by decreased expression of HH components in several preclinical studies [183,184,185]. Considering these promising results, more in vivo studies are necessary in order to verify its efficacy in clinical practice. It is well known from other solid tumors that the initial successful effect of SMO inhibitors is often reduced over time by drug resistance development [10]. Therefore, it is currently postulated to simultaneously inhibit different signaling pathways in order to overcome therapeutic resistance in tumors [186]. Based on the above, the concept of dual targeting of the HH and EGFR pathways in HNSCC has been proposed by several researchers. They reported favorable effects of such combined therapy with the use of HH and EGFR inhibitors in experimental studies [142,187,188]. The dual inhibition of EGFR and HH pathways by cetuximab and vismodegib in combination with cisplatin and docetaxel decreased the cell proliferation and colony-forming ability of HNSCC cells [187]. Likewise, there are studies reporting that blockade of the mTOR pathway increased the effect of HH inhibition in esophageal cancer and HNSCC [183,189].

Recently, interesting results were reported by Kleszcz et al., who presented a combined therapy with inhibitors of several signaling pathways in HNSCC [190]. They analyzed the therapeutic effects caused by selected inhibitors of the following signaling pathways, such as HH (vismodegib), WNT/β-catenin (PRI-724), PI3K (HS-173), and EGFR (erlotinib), separately and in combination on the tongue (CAL 27) and hypopharynx (FaDu) cancer cell lines [190]. The study showed a better response of the hypopharynx than tongue cell lines to the dual inhibition of WNT and HH signaling [190]. A similar effect was observed when simultaneous inhibition with PRI-724 and erlotinib or PRI-724 and the PI3K inhibitor (HS-173) was used [190]. Moreover, the authors postulated that simultaneous inhibition of the WNT and HH pathways decreased CAL 27 and FaDu cell migration and had better anti-proliferative than pro-apoptotic effects. In contrast, PRI-724 combined with erlotinib or HS-173 significantly increased the apoptosis of cell populations, but its individual usage showed lower apoptotic efficacy [190]. Currently, it is widely postulated that a multitherapeutic approach with several anticancer drugs may provide a better response than single therapy, even when molecularly targeted [191]. Therefore, numerous clinical studies in other tumors have been performed that study the efficacy of treatment with HH inhibitors in combination with standard chemotherapy or other targeted therapies [191].

Up to now, only two clinical trials have evaluated SMO inhibitors in HNSCC. The first survey performed by Bowles et al. (ID NCT01255800) studied the combination of IPI-926 (saridegib) with cetuximab in recurrent or metastatic HNSCC patients [192]. This clinical study demonstrated that IPI-926 may be safely combined with cetuximab in HNSCC patients [192]. The dual blockade of EGFR and HH signaling showed tolerable toxicity [192]. Moreover, tumor biopsies during the study revealed that clinical response to therapy was associated with the suppression of EGFR and HH genes [192]. Nonetheless, partial clinical response to treatment was seen only in 12.5% of patients with HNSCC [192]. The authors suggested that inhibition of the HH pathway may increase EGFR signaling, what may indicate that the dosage of IPI-926 enhances response to cetuximab in HNSCC [192]. This observation is in agreement with previous studies showing that HH signaling is a negative regulator of the EGFR signaling pathway and that inhibition of the HH pathway sensitizes tumor cells to EGFR inhibition [142]. On the other hand, in the author’s opinion, the lack of responses in cetuximab-pre-treated patients indicated that HH inhibition may not be enough to convert EGFR-resistant tumors back into EGFR-sensitive tumors. Therefore, HH inhibitors should be used prior to cetuximab in order to increase EGFR-dependent cetuximab sensitivity [192]. However, the experimental data showed that this effect is different in HPV-positive and HPV-negative HNSCCs, and the combination of EGFR and HH inhibition was more effective in HPV-negative tumors [142]. However, the clinical pilot study performed by Bowles et al. had several limitations. The studied group of patients was very limited (*n* = 9) and heterogeneous (different HPV status, cetuximab-naïve, and cetuximab-pre-treated patients). Therefore, the authors concluded that further exploration of this combined therapy is recommended in a more numerous and homogenous cohort [192].

The revealed discrepancy between in vitro and in vivo studies might be a result of both genetic and epigenetic alterations leading to persistent GLI activation that allows tumors to evade inhibition of SMO. This contributes to the development of resistance to therapy and rapid tumor recurrence [191]. A recent report by Yao et al. showed that both JNK/AP-1 and TGFβ/Smad3 pathways simultaneously activate the nuclear myocardin-related transcription factor (nMRTF) that connects with SRF and serves as a coactivator for GLI1 in resistant BCC [193]. It is currently well known that various signaling pathways like WNT, MAPK, NF-κB, PI3K, and TGFβ may activate GLI, what causes that the inhibition of the HH pathway with SMO inhibitors not to be fully effective [191].

The second clinical trial conducted by Mayo Clinic (ID NCT04007744) is still recruiting and has not been completed. This phase I clinical trial evaluates the combined use of sonidegib and pembrolizumab in various tumors, including metastatic HNSCC. However, the results are not yet available.

### 10.4. GLI Inhibitors

The GLI transcription factor is a final protein of the HH pathway, which implies that it is directly involved in HH target gene expression. Therefore, inhibition of GLI seems to be a promising and more effective target for anticancer drugs, contrary to other HH proteins. To date, several GLI inhibitors have been developed, but only a few of them have been tested in vitro in HNSCCs. The most studied GLI antagonist is arsenic trioxide (ATO), which was approved in 2000 by the FDA for the treatment of acute promyelocytic leukemia [194]. However, the anticancer effectiveness of ATO was also tested in solid tumors, including HNSCC [28,195]. The latest in vitro study on OSCC cell lines conducted by Nogueira et al. noted that ATO is a promising cytotoxic agent in OSCC [196]. Treatment with ATO on OSCC cells resulted in reduced GLI1 expression, alterations in the cells’ morphology, nuclear fragmentation, and increased apoptosis [196]. Nonetheless, the main problem is the fact that high doses of ATO are required to effectively inhibit in vivo solid tumors, which in turn causes strong side effects [194]. Based on these findings, current studies are focused on combined therapies with low doses of ATO and other cytotoxic drugs. It was reported in several in vitro studies that ATO may enhance sensitivity to cisplatin and increase their synergistic activity [194,197,198]. Furthermore, other studies suggest that ATO may increase the radiosensitivity of HNSCC [199].

Similar observations were reported in studies evaluating the influence of other GLI inhibitors—GANT61—on OSCC cell lines [200,201]. In vitro experiments showed that GANT61 decreases GLI expression and promotes apoptosis in OSCC cell lines [201]. Likewise, Zubčić et al. observed inhibited proliferation and growth of HNSCC cell lines in response to treatment with GANT61 and lithium chloride, a GSK3β inhibitor [202]. However, none of the GLI inhibitors have been evaluated in clinical studies with the participation of patients suffering from HNSCC. 

## 11. Future Perspectives and Conclusions

To our knowledge, this is the first comprehensive review regarding the HH pathway in HNSCC. We made an effort to collect and summarize recent findings about HH signaling in HNSCC and pointed out the role of this pathway in the biology of HNSCC and its clinical outcome. Based on the latest studies, we may confirm that the HH pathway is aberrantly activated in advanced HNSCC; however, the clinical studies are very limited. As we summarized in this paper, HH signaling is involved in several crucial events during HNSCC oncogenesis, like tumor initiation, neoangiogenesis, cancer invasion, resistance to chemo/radiotherapy, recurrence, and metastasis. Furthermore, increased expression of proteins belonging to the HH pathway is correlated with worse survival and a poor prognosis in HNSCC patients, what may suggest the prognostic significance of HH signaling in HNSCC. Given the present lack of molecularly targeted therapies for patients with HNSCC and low survival rates, inhibition of HH signaling might be a promising intervention in future HNSCC therapies. Therefore, further clinical studies evaluating the efficacy and safety of HH inhibitors in HNSCC treatment are urgently needed. However, due to the strict and complex crosstalk between HH and other signaling pathways, it seems that combined therapy with simultaneous inhibition of several tumorigenic pathways may be required to provide breakthroughs in HNSCC treatment.

## Data Availability

Not applicable.
